# Why Genes Evolve Faster on Secondary Chromosomes in Bacteria

**DOI:** 10.1371/journal.pcbi.1000732

**Published:** 2010-04-01

**Authors:** Vaughn S. Cooper, Samuel H. Vohr, Sarah C. Wrocklage, Philip J. Hatcher

**Affiliations:** 1 Department of Molecular, Cellular, and Biomedical Sciences, University of New Hampshire, Durham, New Hampshire, United States of America; 2 Department of Computer Science, University of New Hampshire, Durham, New Hampshire, United States of America; University of Texas at Austin, United States of America

## Abstract

In bacterial genomes composed of more than one chromosome, one replicon is typically larger, harbors more essential genes than the others, and is considered primary. The greater variability of secondary chromosomes among related taxa has led to the theory that they serve as an accessory genome for specific niches or conditions. By this rationale, purifying selection should be weaker on genes on secondary chromosomes because of their reduced necessity or usage. To test this hypothesis we selected bacterial genomes composed of multiple chromosomes from two genera, *Burkholderia* and *Vibrio*, and quantified the evolutionary rates (*dN* and *dS*) of all orthologs within each genus. Both evolutionary rate parameters were faster among orthologs found on secondary chromosomes than those on the primary chromosome. Further, in every bacterial genome with multiple chromosomes that we studied, genes on secondary chromosomes exhibited significantly weaker codon usage bias than those on primary chromosomes. Faster evolution and reduced codon bias could in turn result from global effects of chromosome position, as genes on secondary chromosomes experience reduced dosage and expression due to their delayed replication, or selection on specific gene attributes. These alternatives were evaluated using orthologs common to genomes with multiple chromosomes and genomes with single chromosomes. Analysis of these ortholog sets suggested that inherently fast-evolving genes tend to be sorted to secondary chromosomes when they arise; however, prolonged evolution on a secondary chromosome further accelerated substitution rates. In summary, secondary chromosomes in bacteria are evolutionary test beds where genes are weakly preserved and evolve more rapidly, likely because they are used less frequently.

## Author Summary

Why many bacteria have multiple chromosomes is largely unknown, but a leading hypothesis is that secondary chromosomes evolved from plasmids and now serve as accessory genomes. We tested a key prediction of this theory that genes on secondary chromosomes should evolve faster because they are under less selective constraint. Indeed, orthologous genes shared within two groups of bacteria (*Burkholderia* or *Vibrio*) with multiple chromosomes were less conserved and evolved more rapidly when found on secondary chromosomes. Much of these patterns could stem from the tendency of secondary chromosomes to be replicated later in the cell cycle, which reduces their gene dosage, their potential for expression, and selection for their optimal translation. However, the content of secondary chromosomes appears to be predisposed to evolve faster, because these same genes still evolve more rapidly in single-chromosome genomes. In summary, the evolution of divided genomes therefore appears to allow for the long-term segregation of genome content by their rates of expression and dispensability, placing some genes at increased risk of mutational decay and greater turnover.

## Introduction

As the number of completely sequenced bacterial genomes has grown, the once surprising discovery of multiple chromosomes has become commonplace. Setting aside the issue of nomenclature (i.e. chromosome or megaplasmid[1]), why some bacterial genomes are divided into multiple, large replicons and others comprised of only a single DNA molecule is largely unknown [2]. Understanding the origin of secondary replicons helps frame the question. Chromosomes may originate by three different mechanisms: by the split of a single chromosome, by chromosome duplication, or by acquisition of a large plasmid with essential genes, which ensures its prolonged maintenance. Of these processes, the last has the greatest support because some secondary chromosomes have plasmid-like origins of replication [2]. However, it is the potential effects of genome subdivision that require further investigation and may explain variation in chromosome number and evolution in bacteria.

One advantage of a divided genome is the potential for faster replication and growth because of multiple origins of DNA replication. For example, *Vibrio* spp. with two chromosomes have among the fastest rates of cell division measured. Yet in all bacteria, the single origin of replication per chromosome means that growth may occur faster than replication, a problem solved by the ability to initiate new cycles of replication before the completion of previous cycles. As a result, daughter cells may be born with multiple partially replicated genomes that are enriched near the origin of replication [3].

Bacteria with multiple chromosomes face the additional challenge of maintaining synchronous replication; if chromosomes are of different sizes, either their timing or their rates of replication must vary. In *Vibrio*, it has been demonstrated that the replication of the smaller, second chromosome is delayed during the cell cycle [4],[5],[6]. This delayed replication in effect reduces the dosage (copy number) of genes on the second chromosome during periods of rapid growth [7], but does not alter the final heredity of each chromosome. Each cell ultimately has one and only one copy of each chromosome (absence of a chromosome would cause it be reassigned as a plasmid), and no evidence yet suggests that this varies. Therefore, variation in how bacterial chromosomes evolve is not, at least given current knowledge, an effect of variation in their effective numbers, as in the sex chromosomes of animals [8].

However, variation in gene dosage during the bacterial cell cycle can have profound effects on the expression of these genes as well as their evolutionary rates. In bacteria with a single chromosome, genes distant from the origin of replication tend to be expressed less than those nearby, and thus distant genes evolve more rapidly [9].

In bacteria with multiple chromosomes, delayed replication of the smaller replicon could produce a similar effect on its expression and thus its evolution. A recent report confirms this effect on expression in fast-growing cells: genes on the late replicating small chromosome of *V. parahaemolyticus* are expressed significantly less than those on the large chromosome, though expression varies more than would be expected from measured dosage effects [4]. In slow growing cells, overlapping replication cycles are unnecessary and hence no dosage and expression bias is found between chromosomes [4]. Replication bias within divided genomes (and particularly those of fast growing species) could therefore accelerate evolution on secondary chromosomes.

This variation in expression caused by genome location, either relative to the origin of replication or on different chromosomes, can in principle exert selection for gene position. Genes that must be expressed frequently should be near the origin of replication and on the primary chromosome [7],[10]. It therefore follows that in *Vibrio*, a significantly greater fraction of growth-essential and growth-contributing genes are found i) on the large, primary chromosome than on the small chromosome, ii) near the origin relative to the terminus of the large chromosome, and even iii) near the terminus of the large chromosome relative to the small chromosome [4]. When grown under optimal conditions, the dosage bias of these genes and hence their expression is exaggerated, but under more limiting conditions dosage bias and expression do not vary with gene position [4],[5]. Moreover, the growth rate *of V. cholerae* slows significantly when the replication rate of the second chromosome is genetically amplified [5],[6]. These findings imply that selection has shaped *Vibrio* genomes to contain genes whose functions benefit from higher dosage during rapid growth on the first chromosome and genes that should be expressed less on the second chromosome [4],[7].

Comparing related genomes with multiple chromosomes also suggests that their content has been segregated by priority and dispensability. In general, the major chromosome tends to have significantly more conserved housekeeping genes, greater overall synteny, and greater conservation of content [7],[11],[12]. Together, these patterns support a general theory that secondary chromosomes are evolutionary test beds subject to reduced purifying selection and thus greater rates of change. The key prediction of this theory is that genes found on secondary chromosomes should evolve faster and more variably than those on the primary chromosome. Furthermore, if genes on secondary chromosomes have been less needed or used over long periods of time, then they should exhibit less bias towards the use of favored synonymous codons (codon usage bias).

We tested this theory by studying the evolutionary rates of ‘panorthologs,’ defined as orthologous genes present in single copy and, for a subset, obeying the consensus species phylogeny, among two sets of monophyletic, completely sequenced genomes with more than one chromosome (*Burkholderia* and *Vibrio*). We then compared the rates of ortholog families found on primary chromosomes with those on secondary chromosomes, calculated the codon bias of these genes, and evaluated their evolutionary patterns in the context of orthologs from sister taxa with only a single chromosome (*Bordetella* and *Xanthomonas*, respectively). We found that orthologs on secondary chromosomes indeed evolved faster and displayed less skew towards purifying selection than those on primary chromosomes. These increased rates of evolution appear to be a consequence of reduced selection for the use of specific codons and translational efficiency because of less frequent expression or necessity [13],[14],[15],[16]. Each prediction of the general theory that secondary chromosomes serve as evolutionary test beds for accessory genes was therefore met.

## Results

### Panorthologs are more numerous and conserved on primary chromosomes

**Figure 1:**
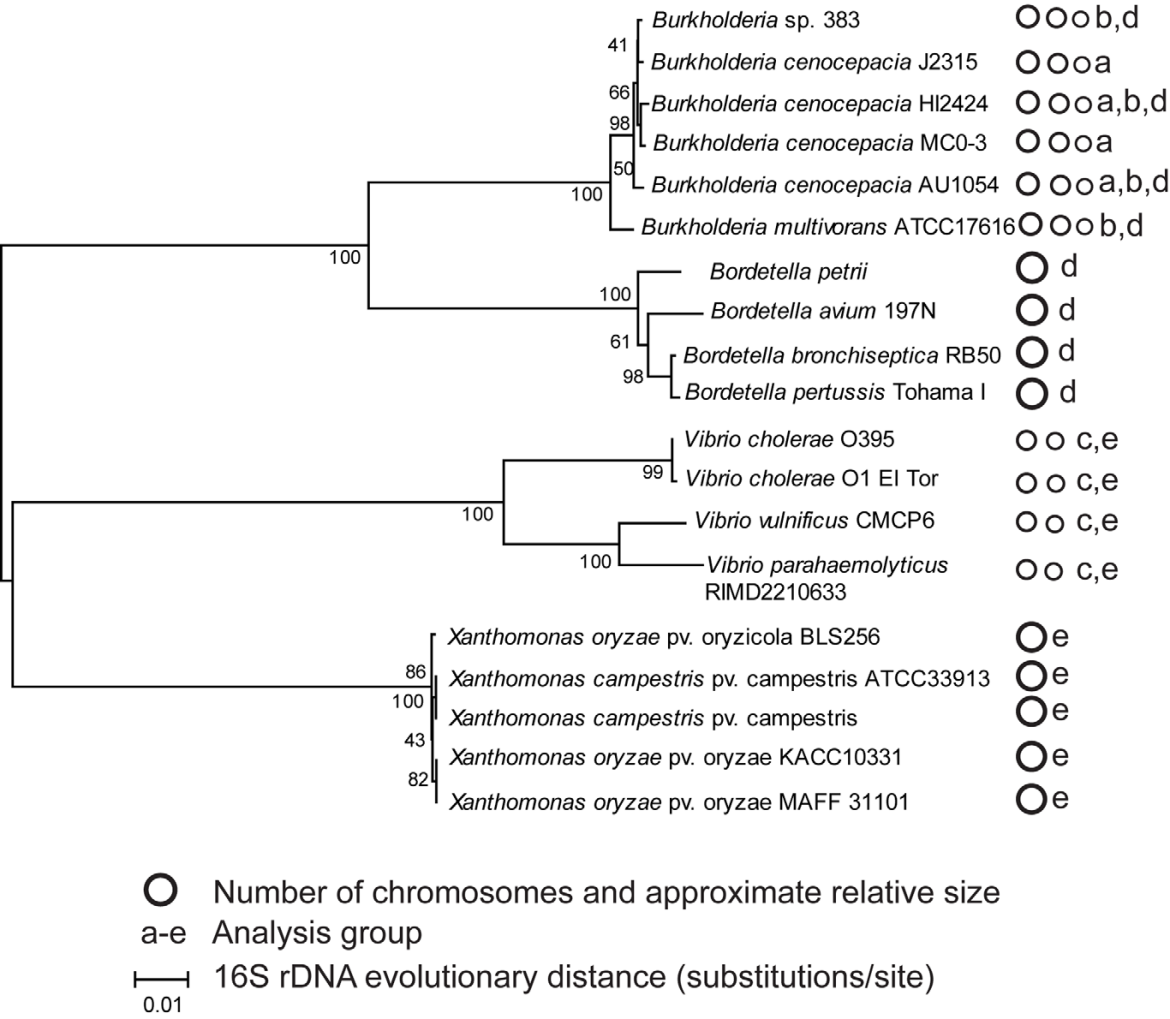
Phylogeny of bacterial genomes studied. Evolutionary history was inferred from complete (1392 bp) 16S sequences by the neighbor-joining method; the bootstrap consensus tree from 500 replicates is shown. Distances were calculated by the maximum composite likelihood method. Analysis was conducted using MEGA4 [46]. Panortholog sets were identified within five genome groups, denoted a–e. *B. cenocepacia* strain PC184 was included in analysis group ‘a’ but its complete 16S sequence was unavailable.

Bacterial genomes with multiple chromosomes were selected from two genera: *Burkholderia* (Beta-Proteobacteria, Burkholderiales, Burkholderiaceae), which have three chromosomes, and *Vibrio* (Gamma-Proteobacteria, Vibrionales, Vibrionaceae), which have two chromosomes. Genomes were selected to span a range of evolutionary distance within each set, from isolates within the same named species to distinct species within the same genus (Fig. 1). This enabled comparisons spanning three different evolutionary distances: i) among strains within the species *B. cenocepacia*, ii) among species within the genus *Burkholderia*, and iii) among more divergent species within the genus *Vibrio*. “Panorthologs,” or orthologs conserved in all genomes within the genome group, were identified by the stringent analysis pipeline described in Methods, based on prior work [16],[17], and discussed in greater detail below. For the remainder of this report we refer to chromosome 1 as c1, chromosome 2 as c2, and chromosome 3 as c3.

**Figure 2:**
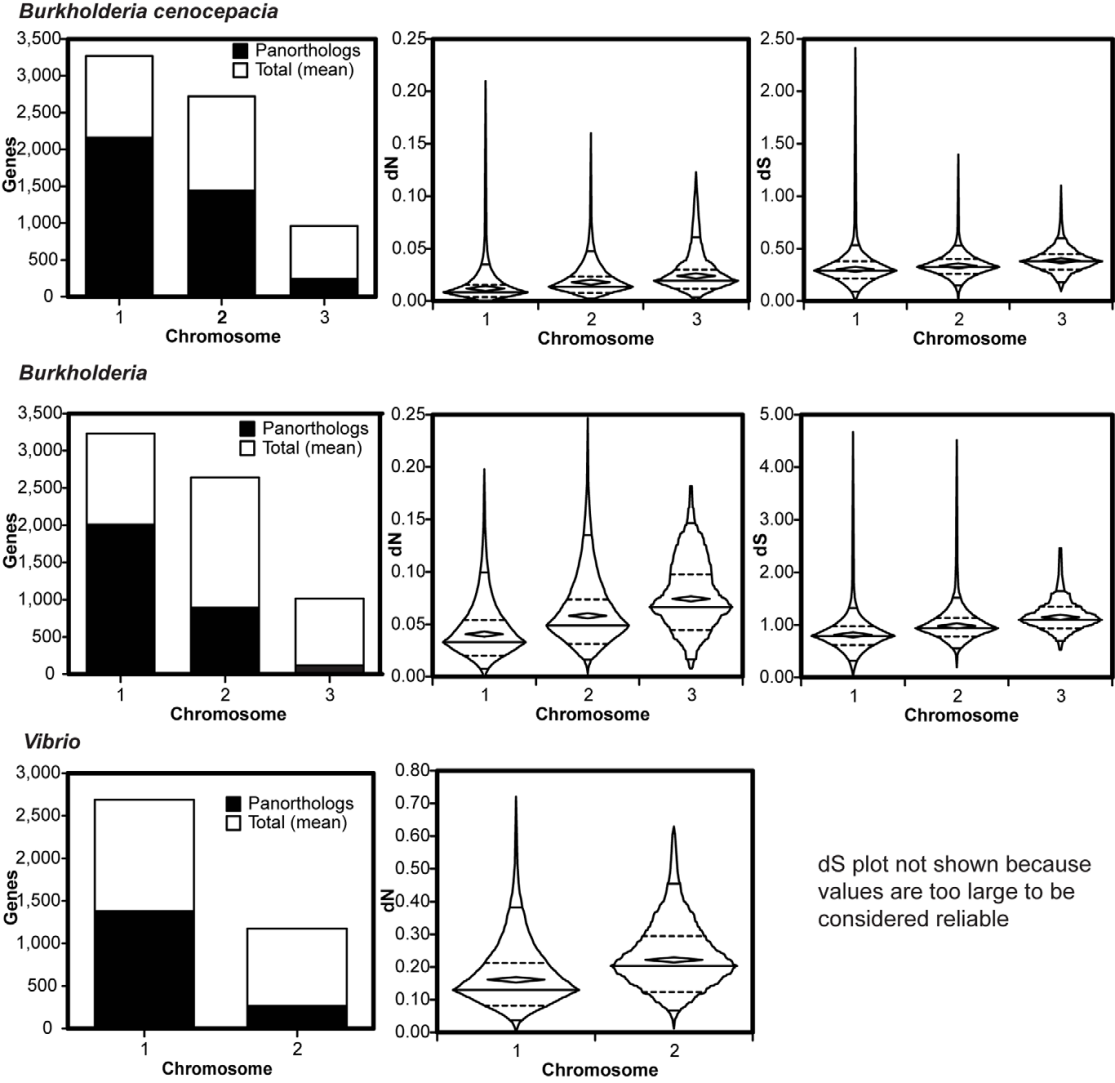
Distribution and evolutionary rates of orthologs vary by chromosome in three sets of bacterial genomes. Orthologs were identified in three different genome sets (Fig. 1, groups a-c) with *B. cenocepacia*, *Burkholderia*, and *Vibrio*. Column 1: orthologs are less abundant on secondary chromosomes, relative to mean genes/chromosome. Panortholog chromosome position was assigned based on the gene position in *B. cenocepacia* HI2424 for groups a and b and *V. cholerae* El Tor N16961 for group c. Columns 2 and 3: the rate of synonymous substitutions per synonymous site (dS) and the rate of nonsynonymous substitutions per nonsynonymous site (dN) among panortholog families both increase significantly on secondary chromosomes (statistical analyses in Tables S1-S3). Figures in columns 2 and 3 are boxplots in which horizontal lines indicate 95th, 75th, 50th, 25th, and 5th percentiles, from top to bottom, interior diamonds indicate the mean, and the exterior shapes represent the overall distribution of the rates on each chromosome.

In each genome collection, panorthologs comprised a lesser fraction of the total genes on secondary chromosomes than on primary chromosomes, and in *Burkholderia*, panorthologs comprised a lesser percentage still on c3 than on c2 (Fig. 2, column 1). This general trend was mostly unaffected by changes in the chromosome position of orthologs within each group. Within *B. cenocepacia*, only 484 of 3848 (12.6%) panorthologs varied in chromosome position, most of which resulted from a large contiguous rearrangement exclusive to the AU1054 genome. The same rearrangement also explained most of the limited variation among *Burkholderia* panorthologs (409 of 2992 varied in chromosome position, or 13.7%). Chromosome positions of panorthologs were also well conserved within *Vibrio*: only of 59 of 1647 (3.6%) varied in chromosome location. In summary, the fraction of panorthologs varied significantly among chromosomes and this finding could not be explained by varied chromosome position among orthologs.

### Rates of synonymous and nonsynonymous substitutions are greater on secondary chromosomes

We began our analysis by quantifying the evolutionary rates of orthologs shared by multiple strains of the same species, *B. cenocepacia*. This group (analysis group A in Fig. 1) provided arguably the most stringent test of our model because minimal evolutionary distance should have accumulated within these gene families; on the other hand, more panorthologs were found in these closely related genomes. Our prediction that evolutionary distance would be greater among orthologs found on secondary chromosomes was met (Fig. 2, Table S1). The distributions of both evolutionary rate parameters, dN and dS, differed among chromosomes, with panorthologs from c2 evolving more quickly than those on c1, and those on c3 more divergent still than those on c2 (Table S1).

We observed the same overall patterns with even greater resolution among different species of *Burkholderia* (Fig. 2, Table S2), even as the total number of panorthologs decreased and as the noise inherent to dN and dS estimates [18] increased. (For this and subsequent analyses, we acknowledge the unreliability of estimates of dS>1 from more divergent homologs; for the different *Burkholderia* species, mean dS only approaches or exceeds 1 on chromosomes 2 and 3.) However, given that these patterns were limited to a particular genus of Beta-Proteobacteria, we sought to test whether chromosome location affected ortholog evolution in different genomes. We chose the genus *Vibrio*, a Gamma-Proteobacteria clade that was one of the first described to harbor multiple chromosomes [19]. Further, we chose more divergent species within *Vibrio* than we had within *Burkholderia* as an additional test (Fig. 1). In studying more divergent genomes we increased the leniency of our ortholog alignments to allow as many as eight consecutive unaligned amino acids instead of the five-site cutoff used within *Burkholderia* (Methods). This produced much greater estimates of dN and dS for *Vibrio* orthologs, the latter being too large to be considered reliable. Nevertheless, we observed essentially the same, statistically significant patterns when comparing the distributions of rates from the two *Vibrio* chromosomes (Fig. 1, Table S3). We note that the fraction of panorthologs on secondary *Vibrio* chromosomes is substantially less than in our *Burkholderia* sets, despite the well-described variability among *Burkholderia* genomes [20].

One of the greatest challenges in phylogenetics is defining orthology [17],[21] and it is possible that our method introduced a systematic bias, so we conducted an even more stringent test of our pipeline. Previously, we included only genes sharing a single, reciprocally best match in all other genomes and whose translated alignment was highly conserved. Here, we also tested whether the panortholog families identified within the five *B. cenocepacia* strains also shared the same strict phylogeny based on branching pattern. Although the number of panortholog families declined substantially due to ambiguous branching (a polytomy) among the J2315, PC184 and MCO-3 genomes, we found the same general patterns (Fig. S1, Table S4). However, this test introduced additional uncertainties because of the number of potential alternative trees (Table S5) and it could be too stringent because different phylogenies could be produced by varying evolutionary rates among lineages. As a result we did not require that all families share the same phylogeny in subsequent analyses, which leaves open the possibility that panortholog families may include genes that vary in their rates of homologous recombination and are not panorthologs in the strictest sense. We return to this issue in Discussion.

We also tested whether using different single genomes within groups to assign panortholog chromosome positions affected our findings. Among the *B. cenocepacia* genomes, using gene positions from the MCO-3 annotation instead of the HI2424 annotation did not alter any interpretations (Table S1). However, the *B. cenocepacia* AU1054 genome contains a unique set of rearrangements between chromosomes 1 and 3 relative to the other *B. cenocepacia* genomes, so we queried the evolutionary rates of these 482 genes in particular. The means and distributions of dN and dS values of these gene families strongly resemble those of their consensus location in the other genomes; that is, genes found on chromosome 1 in all other genomes but found on chromosome 3 in AU1054 are indistinguishable from the other genes on chromosome 1 (F = 0.092, p = 0.762). This suggests that these rearrangements may have occurred recently enough that the chromosome position in AU1054 did not influence the evolutionary rates of their ortholog families.

### Purifying selection is weaker on secondary chromosomes

Perhaps the most telling differences among the rate distributions of each chromosome are their shapes (Fig. 2). In all genome sets, c1 rates exhibited greater positive skew (median < mean) and greater kurtosis than c2 rates, which in turn were more skewed than c3 rates in *Burkholderia* (Table S6). Positive skew and greater kurtosis (observed as greater volume and greater width in the lower half of the shapes in Fig. 2) of rate distributions demonstrate that fast-evolving genes are rarer on c1 than on c2 and c3, even for a given average rate. These properties of the rate distributions are both consistent with purifying selection and suggest that c1 panorthologs are under the greatest selective constraint and those on c2 and c3 are less conserved. In theory, genes may face weaker purifying selection and thus evolve more quickly because they are i) less frequently expressed, which generates less selection for translational efficiency [13],[22],[23] ii) less essential, which should also influence dispensability [16] iii) less connected to multiple functions or pathways [24] or iv) more robust to mutations [25],[26]. Of this incomplete list of explanations, the first has garnered the most comprehensive support [13].

If genes are less frequently expressed and selection for translational accuracy is diminished, then the incorporation of suboptimal codons should be better tolerated. In general, codon usage bias [10],[15],[27] is positively correlated with gene expression [28], although exceptions exist [10],[29]. We estimated codon usage bias using a method based on the Shannon informatics theory and the entropy theory that describes the orderliness of synonymous codon usage (SCUO)[27],[30]. This method facilitates the comparison of codon usage biases both within and across genomes.

**Figure 3:**
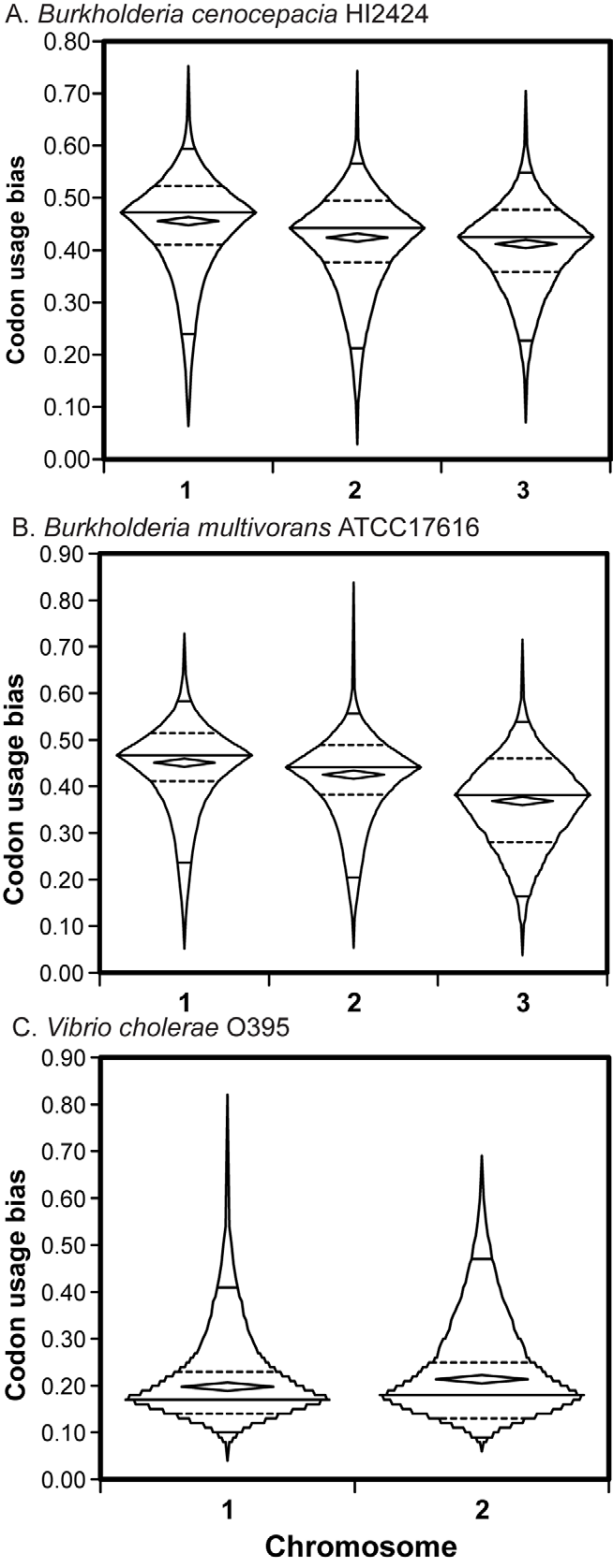
Distributions of synonymous codon usage bias among panorthologs by chromosome location in three representative genomes. Boxplots reflect the overall distribution of SCUO values for all genes on each chromosome; interior diamonds represent the means. All pairwise comparisons are statistically significant (Table 1).

We tested whether genes on secondary chromosomes exhibited systematically less codon usage bias than genes on the primary chromosome in our three genome groups (Fig. 3), and in 11 other genomes with multiple chromosomes (Table 1). Remarkably, in all of these genomes SCUO was significantly less on c2 than on c1, and if applicable, lesser still on c3 than on c2. The distributions of gene codon usage bias also reflected decreased purifying selection on secondary chromosomes; values from c1 genes were significantly more negatively skewed (reflecting stronger bias) than those on c2 or c3 (Fig. 2; skewness of *B. cenocepacia* HI2424 SCUO: c1: −1.02±.044, c2: −0.895±.047, c3: −0.579±.081). Overall codon usage bias varied substantially among genomes and these values associated strongly with their %G+C nucleotide content [27],[31]; the AT-rich *Vibrio* species demonstrated low codon preference values as a result.

**Table 1:** Codon preference (SCUO [27]) is weaker on secondary chromosomes found in all bacterial genomes with multiple chromosomes.

	Chromosome		
Genome	1	2	3
*Burkholderia cenocepacia* HI2424	0.456	0.425	0.411
*B. cenocepacia* AU1054	0.450	0.424	0.435
*B. cenocepacia* J2315	0.453	0.435	0.410
*B. cenocepacia* MCO-3	0.459	0.422	0.389
*B. ambifaria* AMMD	0.453	0.426	0.387
*B*. sp. 383	0.437	0.419	0.371
*B. multivorans* ATCC17616	0.451	0.425	0.369
*B. pseudomallei* K96243	0.455	0.445	
*Vibrio cholerae* El Tor N16961	0.168	0.160	
*V. cholerae* O395	0.168	0.161	
*V. vulnificus* CMP	0.188	0.169	
*V. parahaemolyticus* RIMD	0.213	0.193	
*V. fischeri* ES114	0.269	0.259	
*Agrobacterium tumefaciens* Cereon	0.275	0.243	
*Agrobacterium tumefaciens* DuPont	0.281	0.250	
*Brucella melitensis*	0.256	0.239	
*Ralstonia solanacearum*	0.453	0.413	
*Deinococcus radiodurans*	0.388	0.356	
*Sinorhizobium meliloti*	0.320	0.281	0.232
*Rhodobacter sphaeroides* 2.4.1	0.458	0.443	
*Rhodobacter sphaeroides* ATCC17029	0.462	0.440	
*Silicibacter* TM1040	0.241	0.215	

The distributions of codon usage measurements for each chromosome within each genome were compared by the Kruskal-Wallis test and pairwise comparisons were conducted *post hoc* by Dunn's multiple comparisons test. All comparisons are significant at p<0.002.

To verify our findings that codon bias varied significantly among chromosomes, we also calculated codon usage bias with another set of tools provided by the INteractive Codon usage Analysis (INCA) package [32]. We found that other measures such as the codon adaptation index (CAI) [15] agreed well with SCUO and supported this conclusion (Fig. S2, Table S7). Other metrics (e.g. MELP [32]) that have been shown to reliably infer gene expression as a function of codon usage also predicted that genes on primary chromosomes are expressed more than those on secondary chromosomes (Fig. S2). For *V. cholerae* in particular, the mean CAI was even greater on c1 than c2 that reported by the SCUO method (Table S7). Further, the predicted overall expression levels of *V.cholerae* c1 genes were significantly greater than those on c2 (MELP, c1: 0.495, c2: 0.439, F = 25.6, p<0.0001). Therefore, reduced codon usage bias appears to be an intrinsic attribute of genes on secondary chromosomes, which experience reduced selection for translational efficiency perhaps because of their reduced expression [4] or greater protein dispensability.

### Faster evolutionary rates are both inherent to the genes and affected by chromosome position

**Figure 4:**
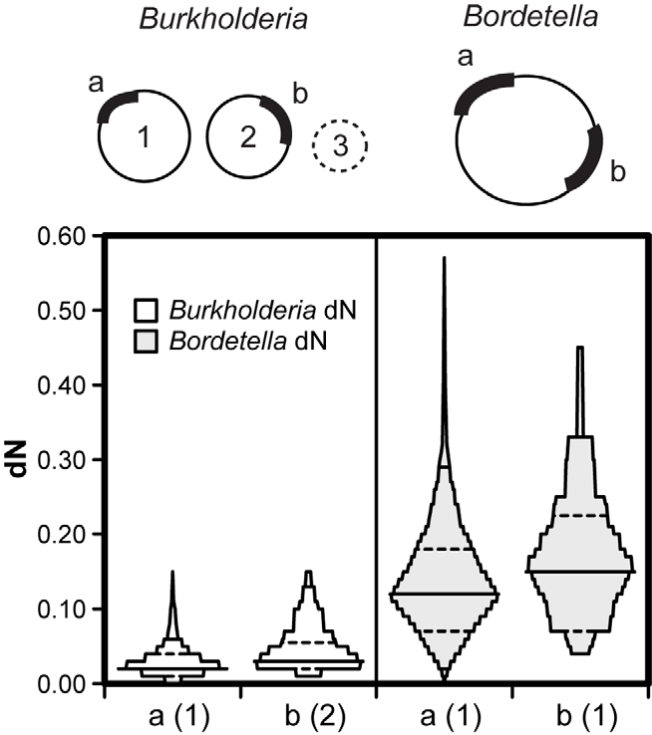
Effects of chromosome position and ortholog identity on evolutionary rates. Primary panorthologs (a, n = 583) are those located on the primary chromosome in multi-chromosome genomes (here, *Burkholderia*) and shared in related genomes with a single chromosome (*Bordetella*). Secondary panorthologs (b, n = 36) are those located on the secondary chromosome in the multichromosome genome. Statistically significant differences in dN among chromosome locations were found both in *Burkholderia* and in *Bordetella* (Table S8). Statistically significant differences in dS between *Burkholderia* chromosomes were also found (Table S8). Mean dN of secondary panorthologs was 56.2% greater than primary panorthologs within *Burkholderia* (effects of both chromosome position and ortholog identity) and 26.1% greater within *Bordetella* (effects of ortholog identity alone).

Relaxed selection on genes found on secondary chromosomes could result from properties of the genes themselves or from general effects of the chromosome, such as delayed replication or reduced copy number that could reduce their likelihood of expression. To discriminate between these possibilities, we identified orthologs shared by multi-chromosome genomes and single-chromosome genomes and quantified their taxon-specific evolutionary rates. We define shared orthologs found on the primary chromosome in the multi-chromosome genomes as “primary panorthologs” and those found on secondary chromosomes as “secondary panorthologs (Fig. 4). Thus, for *Burkholderia* genomes we identified common orthologs in four *Bordetella* genomes (analysis group D, Fig. 1) and for *Vibrio* we found orthologs shared by five *Xanthomonas* genomes (analysis group E, Fig. 1). If relaxed selection is specific to the genes themselves, then secondary panorthologs should evolve more rapidly and demonstrate lesser codon bias than other genes found on the same chromosome, either in *Bordetella* or *Xanthomonas* (Fig. 4). However, if relaxed selection occurs only when orthologs are segregated to a secondary chromosome, then no differences will be found within single-chromosome genomes but significant differences will be found in multi-chromosome genomes. Finally, if both patterns occur but with a greater rate increase within multi-chromosome genomes, then both gene-specific and chromosome-specific processes likely occur.

Among 619 genes shared by *Burkholderia* and *Bordetella* that met the cutoffs of our pipeline (Methods), 583 were primary panorthologs and 36 were secondary panorthologs. The vast difference in their abundances reflects both the dispensability and uniqueness of most genes on secondary chromosomes. We calculated the evolutionary rates of these two groups and found that dN was significantly greater among secondary panorthologs than primary panorthologs in both *Burkholderia* and *Bordetella* (Table S8). Further, fast-evolving orthologs within *Bordetella* were more frequently found on chromosome 2 of *Burkholderia* (Mann-Whitney U = 8348, p = 0.039), and the dN estimates of these genes were less positively skewed (Fig. 4). (We do not present values for dS in this comparison owing to their unreliability (mean dS for *Bordetella* >1.) Together these results suggest that secondary panorthologs inherently evolve faster even when found on the same chromosome, but this effect is magnified by presence on a secondary chromosome. Of the two forces, the effect of chromosome position appears slightly stronger based on our limited evidence. Over the relatively short evolutionary scale separating the *Burkholderia* genomes (Fig. 1), both gene- and chromosome-specific processes could have produced the 56.2% increase in mean dN among secondary panorthologs than primary panorthologs. Among the more divergent *Bordetella* genomes, only gene-specific effects could have generated the 26% increased dN among secondary panorthologs over primary panorthologs (Fig. 4).

We explored the orthologs shared between *Burkholderia* and *Bordetella* for other systematic differences associated with chromosome location. Representatives of the panortholog families found in *B. cenocepacia* HI2424 were used for these analyses. As expected, SCUO was lower among secondary panorthologs, although not significantly so (c1 mean: 0.465, c2 mean: 0.444, F = 2.60, p = 0.11). In addition, the skewness but not the means of the codon adaptation index (CAI) [9] differed between the collections of primary and secondary panorthologs (a negative skew illustrates greater distribution towards highly biased genes; c1 skewness: −0.458±0.11, a significant value, c2 skewness: 0.051±.403, not significant). The most intriguing difference between these two gene sets, however, was their inferred levels of expression (MELP): primary panorthologs were predicted to be expressed significantly more than secondary panorthologs (F = 4.87, p = 0.028). However, the COG annotation of primary and secondary panorthologs did not differ in any obvious manner (Table S9), which suggests that the increased evolutionary rates and lesser expression of secondary panorthologs are not artifacts of an unusual subset of the complete genomes.

Only 99 ortholog families survived our initial filters of orthologs shared between *Vibrio* and *Xanthomonas* (analysis group E, Fig. 1), and only four of these were secondary panorthologs. This group, comprised only of essential genes, was too small to allow us to discriminate between effects of gene or chromosome position. We suspected that the small group resulted from relatively high-quality ortholog alignments within each genus failing to produce a consensus alignment between genera that was not compromised by gaps. To overcome this problem, we included the *V. fischeri* ES114 genome as an intermediate between *Vibrio* and *Xanthomonas* to facilitate more tolerant alignments and to include more panortholog families for analysis. Following this step, we identified 237 orthologs shared between *Vibrio* and *Xanthomonas*, only 13 of which are on the second *Vibrio* chromosome. As we found previously, both dN and dS were significantly greater on the second *Vibrio* chromosome and dN was greater among *Xanthomonas* secondary panorthologs (mean dN = 0.048) than primary panorthologs (mean dN = 0.032), although this difference was not statistically significant (p = 0.089, Table S10). Together, these findings also suggest that evolutionary rate differences are inherent to the genes but are more obviously an effect of chromosome position.

## Discussion

Why some bacterial genomes are composed of multiple chromosomes and others only a single chromosome is a mystery, thought to be a legacy of past plasmid acquisition, entrapment, and genome reshuffling. Yet how bacterial genomes evolve and become subdivided in the aftermath of these events may be quantified using the large number of completely sequenced and annotated bacterial genomes and a well-defined phylogenetic history. With these resources, we tested the theory that secondary chromosomes in bacteria are accessory genomes for specific niches or conditions [10],[12],[33],[34] and thus are evolutionary test beds. The central prediction of this theory is that genes on secondary chromosomes should be subject to weaker purifying selection because of their reduced necessity or usage. Weak purifying selection is manifest as increased evolutionary rates among orthologs (dN and dS), reduced positive skewness of rate distributions from ortholog sets, and reduced codon usage bias. We found that each of these patterns was strongly associated with genes found on secondary chromosomes in three different, phylogenetically independent genome collections from *Burkholderia* and *Vibrio*. Moreover, reduced codon usage bias among genes on secondary chromosomes appears to be a general phenomenon of all multi-chromosome bacteria.

We propose four potential mechanisms that would explain these patterns. First, secondary chromosomes are smaller and so to maintain synchronous replication with the primary chromosome they may be replicated later, as in *Vibrio* [5],[6]. Delayed replication could limit gene copy number within growing cells and systematically minimize expression [7]. Decreased expression should in turn weaken selection for optimal codon usage and increase the synonymous substitution rate, dS, and also reduce selection against protein misfolding because translation events will be fewer and thus increase the nonsynonymous substitution rate, dN [13]. Although we do not measure expression in this study, it has been shown recently that genes on the second chromosome of *V. parahaemolyticus* (a genome included in this study) are expressed less because of delayed replication and reduced dosage [4], and another computational analysis predicts this expression bias in many multi-chromosome genomes [7].

Second, a defining feature of secondary chromosomes is their relative rarity of orthologs conserved among related genomes (Fig. 2), which implies that these genes are more dispensable. This dispensability is not the property referred to in previous studies of the correlates of evolutionary rates (e.g. [16]), effects of experimental gene knockouts, but rather their likelihood to be lost following speciation. Genes that are more dispensable should be under weaker purifying selection, in general, and both dS and dN should increase. Further, if selection against protein misfolding is as strong as has been argued [13], the deleterious effects of misfolded proteins could generate positive selection for their deletion. Exactly why these genes are or become more dispensable has prompted much speculation: secondary chromosomes have been thought to be niche-specific and thus only conditionally useful in dynamic environments [12], which could cause genes on secondary chromosomes to be lost frequently by drift (because they are useless) or by antagonistic pleiotropy (because they now reduce fitness) [35]. Of these two forces, gene loss driven by selection is almost certainly more rapid. When we inspect the evolution of the content of divided genomes over a relatively short time span (e.g. closely related strains of *B. cenocepacia* and species of *Burkholderia* (Fig. 2)), we find that most differences occur on secondary chromosomes. Given that such species likely have very large effective population sizes that minimize effects of drift relative to selection [36], we suggest that selection for the loss of orthologs explains why such genes are weakly preserved on secondary chromosomes.

The differential gene preservation among primary and secondary chromosomes could also shed light on the relative roles of selection and drift in gene rearrangement. Those orthologs that persist on secondary chromosomes for long evolutionary periods become noteworthy given their generally high loss rate. If these remaining orthologs have been preserved by selection and not just by chance, then their initial rearrangement to a secondary chromosome could have been favored. Our analysis of orthologs shared by genomes with multiple chromosomes and those with one chromosome supports this model, as genes that relocated to the secondary chromosome evidently already evolved more rapidly (Fig. 4), were less codon-adapted, and are predicted to be expressed less even when confined to a single chromosome. We acknowledge, however, that gene relocation to secondary chromosomes is a chicken-and-egg problem: which came first, selection for reduced expression or an increase in dispensability that caused relocation to be selectively neutral? We speculate that differential expression among genome locations presents a means for selection to tune the activity of individual genes by relocating them either nearer the replication terminus of the primary chromosome, or when they are present, to secondary chromosomes. As such gene rearrangements are probably more rare than other mutations that alter expression (e.g. SNPs in regulatory sequences), however, positive selection for rearrangement is also likely rare. Regardless, the long-term effect of these rearrangements, driven initially by either drift or selection, is greater evolutionary rates.

A third mechanism that could explain the patterns presented here is that secondary chromosomes may be inherently more tolerant and/or more prone to recombination of homologous alleles. Increased homologous recombination of divergent alleles would generate many of the patterns reported here and offers an alternative interpretation of our findings. We disfavor this interpretation because recombination should reduce similarity and greatly decrease the probability that genes in different lineages will meet our stringent tests for homology and orthology (Methods). However, to test this alternative, we recognized that recombination should create incongruent phylogenies among genomes and analyzed only those ortholog families sharing the consensus phylogeny. Of the genome sets presented here, the collection of different strains of *B. cenocepacia* provides the most rigorous test, as lineages within the same species are expected to have undergone recombination more frequently than different species. Thus we analyzed only those panorthologs that conformed to the strict consensus phylogenetic topology within the *B. cenocepacia* genomes, and this subset still demonstrated both significantly increased and less skewed rates of evolution among genes on secondary chromosomes. However, we did not subject the other genome sets to this analysis and acknowledge that their panorthologs could demonstrate effects of recombination on inferred evolutionary rates.

A fourth possible mechanism is that secondary chromosomes could experience inherently higher mutation rates. Although mutation rates are known to vary among genome locations, such a widespread and systematic difference would be exceptional. The delayed replication of secondary chromosomes could potentially produce such an effect if nucleotide pools vary or become limiting as a function of the cell cycle [37] or if the replication apparatus tends to require reassembly in later replication stages, which is mutagenic [38]. The probable origin of secondary chromosomes as plasmids could also lead to increased mutation rates as a consequence of their greater supercoiling, which has been associated with greater rates of mutation [39]. Of the four potential explanations that we suggest for why secondary chromosomes evolve more quickly, this one (a systematically greater mutation rate) is the most speculative but also the most experimentally tractable.

It is inevitable that even more powerful studies of the effect of multiple chromosomes on evolutionary rates of bacterial genes will be possible as more complete genomes become available. It may be possible to compare evolutionary rates among distinct taxa of equivalent internal phylogenetic distance, which may allow us to better isolate the effect of chromosome addition. Implementation of more systematic studies of phylogenetic branch length as well as topology could also improve ortholog detection. Our design here was optimized for the genomes available at the time and we compared evolutionary rates of orthologs shared by neighboring taxa (e.g. between *Burkholderia* and *Bordetella*) with caution, given the many factors that could influence relative rates.

However, if the generally increased evolvability of secondary chromosomes holds true for most or all multipartite bacterial genomes, we may be able to better understand how genomes evolve and function. First, simply finding that genes are located on smaller secondary chromosomes may indicate their selection for reduced use or their dispensability. If orthologs of these genes are found in related genomes and in a conserved location, then their products may be optimally expressed at lower levels; if absent, then they are more likely dispensable. Second, reduced purifying selection on secondary chromosomes should accelerate divergence among multipartite genomes in general. Given current species definitions based on empirical measures of DNA similarity or average nucleotide identity [20],[40], bacterial taxa comprised of multiple chromosomes will apparently be more prone to speciate because of the greater divergence of secondary chromosomes. These predictions are confirmed within the *Burkholderiacae*, which display unusually high genomic diversity for a given level of divergence in 16S rDNA sequence [20],[41]; further, most of this genome divergence is found on secondary chromosomes (Fig. 2).

We anticipate the need for more focused analyses of the nature of highly evolvable genes and chromosomes, including their associations with certain functions, their levels of expression during the cell cycle, and their broader membership within homologous gene families. If one way for bacteria to control the magnitude of gene expression is related to gene location, then genes that should be expressed minimally or late in the cell cycle could be selected for relocation distant from the replication origin or on secondary chromosomes. However, we speculate that this could introduce a life-history tradeoff within the genome for such functions, as they would be expected to evolve more rapidly owing to weaker purifying selection for efficient translation. Such a tradeoff is analogous to the origins of senescence, in which genes required early in life and concurrently with reproduction are under strong selection whereas those used past the age of reproduction are more prone to decay and are more dispensable. In summary, secondary chromosomes in bacteria appear to occupy a netherworld between the conserved, core genome found mostly on primary chromosomes and the transiently necessary accessories found on plasmids, offering the benefits and costs of both.

## Methods

### Genomes and definitions

Annotations of bacterial genomes were downloaded from the Integrated Microbial Genome database (IMG; http://img.jgi.doe.gov) in FASTA nucleotide and amino acid formats for each chromosome. Chromosomes were defined as primary or secondary based on their annotation; in all genomes studied but one, chromosome number is defined in decreasing order of size. The one exception was the *V. cholerae* O395, in which c2 and c1 definitions were reversed relative to the annotations of all other *Vibrio*.

### Codon usage preference

We calculated codon preference using a method based on Shannon information theory and entropy theory described by Wan et al. [27],[30]. The metric, SCUO, was calculated using the CodonO software [30]. Gene annotations for each chromosome were analyzed using this method and values for each gene were retrieved. Codon bias measures for each chromosome were then compared by ANOVA and by Kruskal-Wallis tests as described. To calculate CAI [15] and MELP[42], we downloaded genes encoding ribosomal proteins for each analyzed genome to serve as a reference for codon preference. This reference file and the complete annotations for each chromosome were uploaded into the INCA software [42], codon preference was calculated for each gene, and then the measures for each chromosome were compared by ANOVA.

### Identification of panorthologs

We began computation of putative panorthologs for each set of genomes using NCBI BLASTP (release 2.2.16) to analyze all genes in all genomes for sequence similarity. We kept for later processing all BLAST hits within an E-value threshold of 1. These hits include each gene's self hit. We stored the E-value, bit score and alignment length for each hit. When running BLASTP, we used default parameters except for setting the E-value threshold and for setting the maximum number of hits to keep.

We next identified homologs as those gene pairs that had BLAST hits in both directions within a given scaled bit score threshold. We scaled the bit scores by the bit score of the self hit of the query gene. That is, scaledBitScore(A->B) = bitScore(A->B)/bitScore(A->A). This method has been used previously to identify conserved homologs among bacterial genomes and has been shown to be more stringent than criteria based solely on reciprocal best matches using E values [17].

We then formed homolog families by including two genes in a family if they had been identified as homologs. Note that not all pairs of genes in a family need to be identified as homologs. For example, if A and B are homologs, and B and C are homologs, then A and C will be in the same family even if A and C have not been identified as homologs. Finally we identified the putative panorthologs as being the genes from homolog families with exactly one gene from each genome. For each set of genomes we kept the largest set of panorthologs found by computing the putative panorthologs while varying the scaled bit score threshold from .1 to .9 in .1 increments.

The following scaled bit score thresholds were used for genome sets A–E depicted in Fig. 1, followed by the number of putative panorthologs identified at that threshold: group A: threshold = 0.7, 4141 panorthologs; group B: 0.7, 3758, group C: 0.4, 2203, group D: 0.3, 902, group E: 0.2, 581. To produce groups d and e, the five *Bordetella* genomes were first analyzed by this method (0.5, 1592) as well as the five *Xanthomonas* genomes (0.5, 2450). The intersections of these *Bordetella* and *Xanthomonas* panortholog sets with groups b and c were used to produce groups d and e, respectively.

### Measurements of evolutionary rates

We developed a pipeline analogous to the one described by Wall et al [16]. The amino acid sequences of each putative panortholog family was first aligned using ClustalW2 [43]. Next, we used the codon boundaries to align the nucleotide sequences. The leading and trailing edges of each amino acid sequence in every family was trimmed to generate consensus edges, and then the nucleotide sequences were trimmed to match. From this trimmed file, a consensus sequence for the family was found, using the cons utility from the EMBOSS suite. Each sequence in the family was compared against the consensus sequence and if any gene in the family differed from the consensus by more than the specified threshold number of amino acid differences, the family was discarded from further analysis. The following are the amino acid alignment thresholds used for each genome group: group A: five amino acids, group B: five, group C: eight, group D: eight, group E: eight.

Phylogenetic trees were then constructed for each family using DNAML (maximum likelihood) in PHYLIP [44] using default settings and the Newick formatted trees were saved. Finally, dN and dS were calculated from the trimmed nucleic acid alignment and the DNAML tree as a guide using codeml in the PAML package [45]. Codeml model 0, which allows for a single dN and dS value throughout the phylogeny, was used.

In calculating evolutionary rates of panorthologs shared by two sets of organisms (e.g. *Burkholderia* and *Bordetella*), we aligned all taxa in both families, trimmed their edges and discarded families with excessive gaps, but then separated these genes back into their genus groups for analysis by PHYLIP/dnaml and PAML/codeml. This produces dN and dS values for each group within these larger panortholog sets rather than just a single value.

## Supporting Information

Figure S1 Evolutionary rates among panorthologs that shared a strict consensus phylogeny among strains of *Burkholderia cenocepacia* (complete results in Table S5). Shapes are boxplots in which horizontal lines indicate 95th, 75th, 50th, 25th, and 5th percentiles, from top to bottom, interior diamonds indicate the mean, and the exterior shapes represent the overall distribution of the rates on each chromosome. Both dN and dS decline significantly with increasing chromosome number (Table S4).
(0.22 MB TIF)

Figure S2 Codon adaptation index (CAI) and predicted level of expression (MELP) of genes found on different chromosomes of A. *B. cenocepacia* HI2424 and B. *V. cholerae* El Tor N16961.
(0.40 MB TIF)

Table S1 Analyses of variance (ANOVA) among evolutionary rates (dN and dS) within *Burkholderia cenocepacia* by chromosome location.
(0.05 MB DOC)

Table S2. ANOVA among evolutionary rates (dN and dS) within *Burkholderia* by panortholog chromosome location.
(0.04 MB DOC)

Table S3 ANOVA of the evolutionary rate dN among *Vibrio* genomes by panortholog chromosome location. dS analysis was omitted because of unreliably high estimates (means >1).
(0.03 MB DOC)

Table S4 Analysis of distributions of evolutionary rates among panorthologs within *Burkholderia cenocepacia* sharing a common phylogeny of (((J2315,PC184),MCO-3),AU1054,HI2424).
(0.04 MB DOC)

Table S5 Alternative phylogenies of panorthologs identified in *B. cenocepacia* strains HI2424, AU1054, MCO-3, PC184, and J2315.
(0.03 MB DOC)

Table S6 Skewness and Kurtosis (± SE) of distributions of dN and dS measurements from each panortholog set (groups a-e as in Figure 1). Skewness and kurtosis are considered significant if the ratio of the coefficient to its standard error is greater than 2. All distributions except those denoted with an asterisk (*) are significantly skewed or peaked. Smaller coefficients suggest weaker purifying selection as the median approaches the mean.
(0.04 MB DOC)

Table S7 Different measures of codon usage bias and predicted expression among genes on different chromosomes (c1-c3).
(0.04 MB DOC)

Table S8 Analyses of variance among evolutionary rates of primary and secondary panorthologs shared between *Burkholderia* and *Bordetella*. *Bordetella* dS results were omitted because they are too high to be reliable.
(0.04 MB DOC)

Table S9 Distribution of panorthologs shared by *Burkholderia* and *Bordetella* by chromosome location in *Burkholderia* and COG annotation.
(0.05 MB DOC)

Table S10 Analyses of variance of the rates of nonsynonymous substitutions among primary and secondary panorthologs shared between *Vibrio* and *Xanthamonas*. Estimates of rates of synonymous substitutions were omitted because they are too high to be considered reliable.
(0.04 MB DOC)
