# Supplementary figures and images for: Why Genes Evolve Faster on Secondary Chromosomes in Bacteria
Source: PLoS Comput Biol. 2010 Apr 1;6(4):e1000732. doi: 10.1371/journal.pcbi.1000732 (PMC2848543; doi:10.1371/journal.pcbi.1000732)

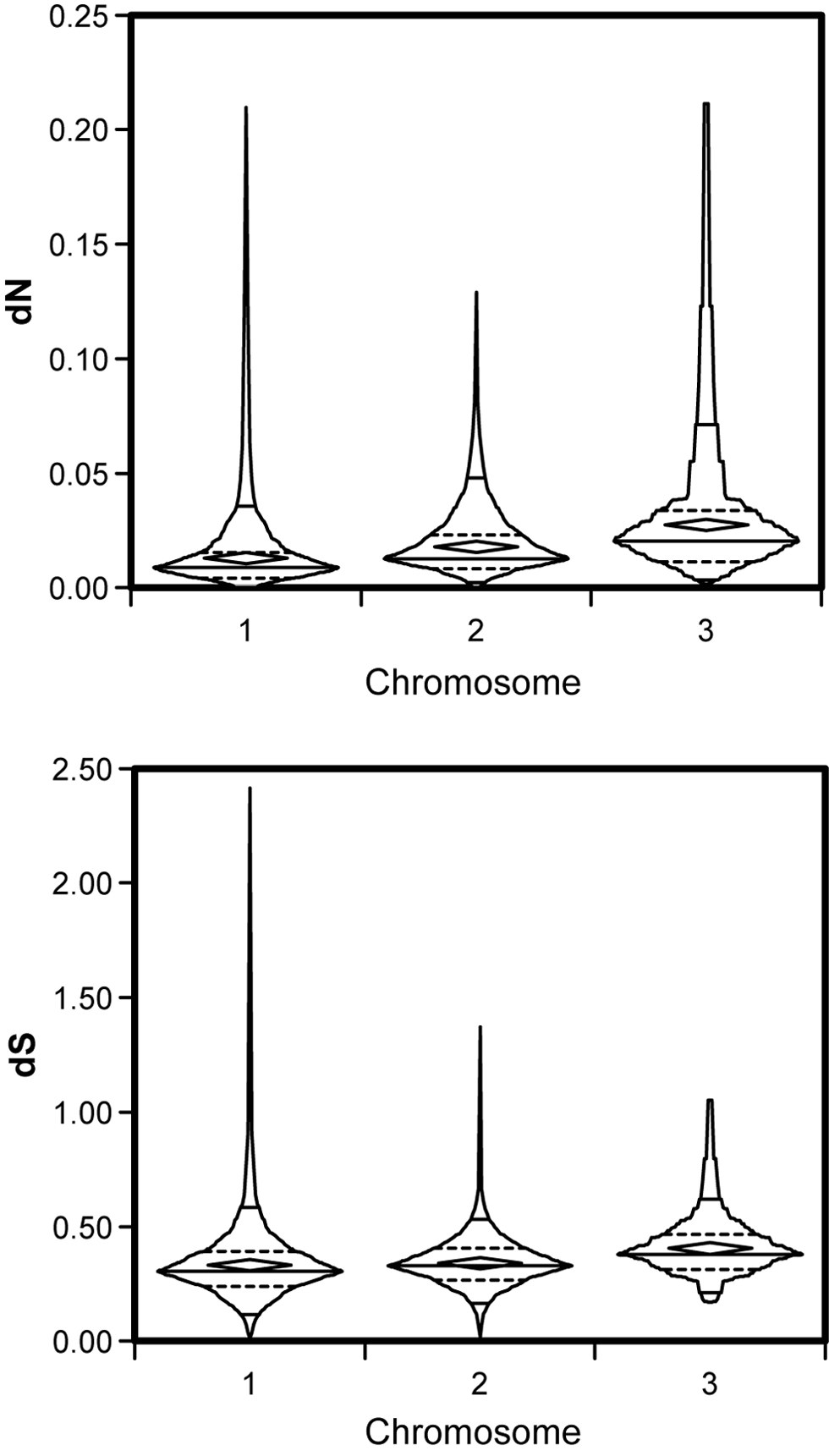

Supplement: Figure S1 — Evolutionary rates among panorthologs that shared a strict consensus phylogeny among strains of Burkholderia cenocepacia (complete results in Table S5). Shapes are boxplots in which horizontal lines indicate 95th, 75th, 50th, 25th, and 5th percentiles, from top to bottom, interior diamonds indicate the mean, and the exterior shapes represent the overall distribution of the rates on each chromosome. Both dN and dS decline significantly with increasing chromosome number (Table S4). (0.22 MB TIF) [file pcbi.1000732.s001.tif]

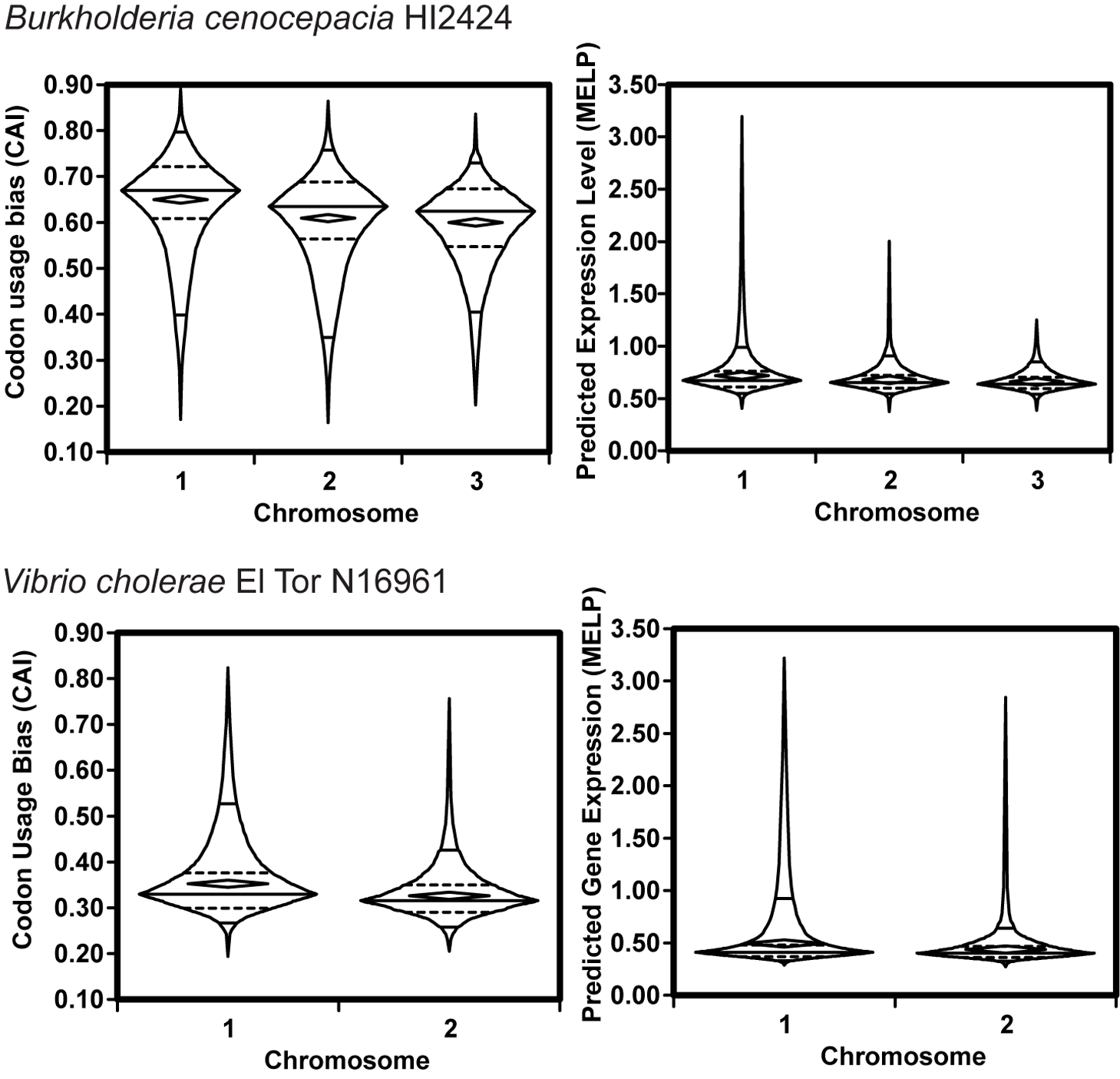

Supplement: Figure S2 — Codon adaptation index (CAI) and predicted level of expression (MELP) of genes found on different chromosomes of A. B. cenocepacia HI2424 and B. V. cholerae El Tor N16961. (0.40 MB TIF) [file pcbi.1000732.s002.tif]
