# Supplementary material for: Why Genes Evolve Faster on Secondary Chromosomes in Bacteria
Source: PLoS Comput Biol. 2010 Apr 1;6(4):e1000732. doi: 10.1371/journal.pcbi.1000732 (PMC2848543; doi:10.1371/journal.pcbi.1000732)
Supplement: Table S1 — Analyses of variance (ANOVA) among evolutionary rates (dN and dS) within Burkholderia cenocepacia by chromosome location. (0.05 MB DOC) [file pcbi.1000732.s003.doc]

Table S1. Analyses of variance (ANOVA) among evolutionary rates (dN and dS) within *Burkholderia cenocepacia* by chromosome location.

a. Chromosome location of panortholog from *B. cenocepacia* HI2424 annotation

|  |  | Sum of squares | df | Mean square | F | Significance |
| --- | --- | --- | --- | --- | --- | --- |
| dN | Between chromosomes | .053 | 2 | .027 | 120.88 | <.0001 |
|  | Within chromosomes | .849 | 3845 | .000 |  |  |
|  | Total | .902 | 3847 |  |  |  |
| dS | Between chromosomes | 1.955 | 2 | .978 | 46.479 | <.0001 |
|  | Within chromosomes | 80.882 | 3845 | .021 |  |  |
|  | Total | 82.837 | 3847 |  |  |  |

b. Chromosome location of panortholog from *B. cenocepacia* MCO-3 annotation

|  |  | Sum of squares | df | Mean square | F | Significance |
| --- | --- | --- | --- | --- | --- | --- |
| dN | Between chromosomes | .053 | 2 | 0.027 | 121.0 | <.0001 |
|  | Within chromosomes | .848 | 3843 | .000 |  |  |
|  | Total | .902 | 3845 |  |  |  |
| dS | Between chromosomes | 1.988 | 2 | .994 | 47.64 | <.0001 |
|  | Within chromosomes | 80.18 | 3843 | .021 |  |  |
|  | Total | 83.17 | 3845 |  |  |  |
