# Supplementary material for: Why Genes Evolve Faster on Secondary Chromosomes in Bacteria
Source: PLoS Comput Biol. 2010 Apr 1;6(4):e1000732. doi: 10.1371/journal.pcbi.1000732 (PMC2848543; doi:10.1371/journal.pcbi.1000732)
Supplement: Table S2. — ANOVA among evolutionary rates (dN and dS) within Burkholderia by panortholog chromosome location. (0.04 MB DOC) [file pcbi.1000732.s004.doc]

Table S2. Analyses of variance (ANOVA) among evolutionary rates (dN and dS) within *Burkholderia* by panortholog chromosome location.

|  |  | Sum of squares | df | Mean square | F | Significance |
| --- | --- | --- | --- | --- | --- | --- |
| dN | Between chromosomes | .260 | 2 | .130 | 122.19 | <.0001 |
|  | Within chromosomes | 3.179 | 2989 | .001 |  |  |
|  | total | 3.439 | 2991 |  |  |  |
| dS | Between chromosomes | 24.804 | 2 | 12.40 | 111.76 | <.0001 |
|  | Within chromosomes | 331.69 | 2989 | .111 |  |  |
|  | total | 356.50 | 2991 |  |  |  |
