# Supplementary material for: Why Genes Evolve Faster on Secondary Chromosomes in Bacteria
Source: PLoS Comput Biol. 2010 Apr 1;6(4):e1000732. doi: 10.1371/journal.pcbi.1000732 (PMC2848543; doi:10.1371/journal.pcbi.1000732)
Supplement: Table S3 — ANOVA of the evolutionary rate dN among Vibrio genomes by panortholog chromosome location. dS analysis was omitted because of unreliably high estimates (means >1). (0.03 MB DOC) [file pcbi.1000732.s005.doc]

Table S3. ANOVA of the evolutionary rate dN among *Vibrio* genomes by panortholog chromosome location. dS analysis was omitted because of unreliably high estimates (means > 1).

|  |  | Sum of squares | df | Mean square | F | Significance |
| --- | --- | --- | --- | --- | --- | --- |
| dN | Between chromosomes | 1.280 | 1 | 1.28 | 72.90 | <.0001 |
|  | Within chromosomes | 28.88 | 1645 | 0.18 |  |  |
|  | total | 30.16 | 1646 |  |  |  |
