# Supplementary material for: Why Genes Evolve Faster on Secondary Chromosomes in Bacteria
Source: PLoS Comput Biol. 2010 Apr 1;6(4):e1000732. doi: 10.1371/journal.pcbi.1000732 (PMC2848543; doi:10.1371/journal.pcbi.1000732)
Supplement: Table S4 — Analysis of distributions of evolutionary rates among panorthologs within Burkholderia cenocepacia sharing a common phylogeny of (((J2315,PC184),MCO-3),AU1054,HI2424). (0.04 MB DOC) [file pcbi.1000732.s006.doc]

Table S4. Analysis of distributions of evolutionary rates among panorthologs within *Burkholderia* *cenocepacia* sharing a common phylogeny of (((J2315,PC184),MCO-3),AU1054,HI2424). Owing to uneven and statistically distinct distributions of evolutionary rates, we used a nonparametric Kruskal-Wallis test rather than a standard ANOVA.

|  | Chromosome | N | Mean rank |
| --- | --- | --- | --- |
| dN | 1 | 551 | 473.5 |
|  | 2 | 501 | 627.9 |
|  | 3 | 66 | 758.8 |
| dS | 1 | 551 | 522.3 |
|  | 2 | 501 | 577.9 |
|  | 3 | 66 | 729.9 |
|  | Total | 1118 |  |

Test statistics:

|  | dN | dS |
| --- | --- | --- |
| Chi-square | 86.73 | 27.30 |
| df | 2 | 2 |
| Asymptotic significance | <.0001 | <.0001 |
