# Supplementary material for: Why Genes Evolve Faster on Secondary Chromosomes in Bacteria
Source: PLoS Comput Biol. 2010 Apr 1;6(4):e1000732. doi: 10.1371/journal.pcbi.1000732 (PMC2848543; doi:10.1371/journal.pcbi.1000732)
Supplement: Table S5 — Alternative phylogenies of panorthologs identified in B. cenocepacia strains HI2424, AU1054, MCO-3, PC184, and J2315. (0.03 MB DOC) [file pcbi.1000732.s007.doc]

Table S5. Alternative phylogenies of panorthologs* identified in *B. cenocepacia* strains HI2424, AU1054, MCO-3, PC184, and J2315.

(((AU1054,HI2424),J2315),MCO-3,PC184);  256
((HI2424,MCO-3),(J2315,PC184),AU1054);  26
((AU1054,HI2424),(J2315,MCO-3),PC184);  15
((AU1054,PC184),(HI2424,J2315),MCO-3);  1
(((AU1054,PC184),J2315),HI2424,MCO-3);  3
((AU1054,J2315),(MCO-3,PC184),HI2424);  17
((AU1054,MCO-3),(HI2424,J2315),PC184);  2
(((AU1054,MCO-3),HI2424),J2315,PC184);  1
((AU1054,J2315),(HI2424,MCO-3),PC184);  1
(((HI2424,PC184),AU1054),J2315,MCO-3);  2
(((J2315,MCO-3),AU1054),HI2424,PC184);  24
(((J2315,PC184),HI2424),AU1054,MCO-3);  58
((AU1054,J2315),(HI2424,PC184),MCO-3);  3
((HI2424,J2315),(MCO-3,PC184),AU1054);  9
(((AU1054,HI2424),MCO-3),J2315,PC184);  30**
(((AU1054,HI2424),PC184),J2315,MCO-3);  23
((AU1054,MCO-3),(J2315,PC184),HI2424);  53
((AU1054,HI2424),(MCO-3,PC184),J2315);  2
((AU1054,PC184),(J2315,MCO-3),HI2424);  16
(((MCO-3,PC184),HI2424),AU1054,J2315);  8
(((HI2424,MCO-3),AU1054),J2315,PC184);  1
((AU1054,HI2424),(J2315,PC184),MCO-3);  40**
(((HI2424,MCO-3),J2315),AU1054,PC184);  3
(((J2315,PC184),MCO-3),AU1054,HI2424);  1049**
(((J2315,MCO-3),PC184),AU1054,HI2424);  1052
(((AU1054,J2315),MCO-3),HI2424,PC184);  11
(((HI2424,J2315),AU1054),MCO-3,PC184);  7
((HI2424,PC184),(J2315,MCO-3),AU1054);  3
(((AU1054,MCO-3),J2315),HI2424,PC184);  3
(((J2315,PC184),AU1054),HI2424,MCO-3);  98
(((HI2424,MCO-3),PC184),AU1054,J2315);  2
(((HI2424,J2315),MCO-3),AU1054,PC184);  2
(((AU1054,MCO-3),PC184),HI2424,J2315);  6
(((HI2424,J2315),PC184),AU1054,MCO-3);  8
(((AU1054,J2315),HI2424),MCO-3,PC184);  9
(((AU1054,PC184),MCO-3),HI2424,J2315);  2
(((J2315,MCO-3),HI2424),AU1054,PC184);  18
(((MCO-3,PC184),J2315),AU1054,HI2424);  973
(((MCO-3,PC184),AU1054),HI2424,J2315);  12
(((HI2424,PC184),J2315),AU1054,MCO-3);  6
(((AU1054,J2315),PC184),HI2424,MCO-3);  6

*Panorthologs were identified as described in Materials and Methods with an alignment cutoff of 5 amino acids. Phylogenies were calculated using default settings of DNAML [1]. **Equivalent phylogenies comprising the consensus, n=1119.

1. Felsenstein J (1989) PHYLIP - Phylogeny Inference Package (Version 3.2). Cladistics 5: 164 - 166.
