# Supplementary material for: Why Genes Evolve Faster on Secondary Chromosomes in Bacteria
Source: PLoS Comput Biol. 2010 Apr 1;6(4):e1000732. doi: 10.1371/journal.pcbi.1000732 (PMC2848543; doi:10.1371/journal.pcbi.1000732)
Supplement: Table S6 — Skewness and Kurtosis (± SE) of distributions of dN and dS measurements from each panortholog set (groups a-e as in Figure 1). Skewness and kurtosis are considered significant if the ratio of the coefficient to its standard error is greater than 2. All distributions except those denoted with an asterisk (*) are significantly skewed or peaked. Smaller coefficients suggest weaker purifying selection as the median approaches the mean. (0.04 MB DOC) [file pcbi.1000732.s008.doc]

Table S6. Skewness and Kurtosis (± SE) of distributions of dN and dS measurements from each panortholog set (groups a-e as in Figure 1). Skewness and kurtosis are considered significant if the ratio of the coefficient to its standard error is greater than 2. All distributions except those denoted with an asterisk (*) are significantly skewed or peaked. Smaller coefficients suggest weaker purifying selection as the median approaches the mean.

Group a.

|  |  | Chromosome 1 | Chromosome 2 | Chromosome 3 |
| --- | --- | --- | --- | --- |
| dN | Skewness | 3.22 (.053) | 1.58 (.064) | 1.07 (.156) |
|  | Kurtosis | 32.31 (.105) | 9.39 (.129) | 3.90 (.311) |
| dS | Skewness | 4.09 (.053) | 2.35 (.064) | 2.27 (.156) |
|  | Kurtosis | 34.04 (.105) | 9.89 (.129) | 6.72 (.311) |

Group b.

|  |  | Chromosome 1 | Chromosome 2 | Chromosome 3 |
| --- | --- | --- | --- | --- |
| dN | Skewness | 1.60 (.055) | 1.52 (.082) | .638 (.254) |
|  | Kurtosis | 3.50 (.109) | 2.86 (.164) | -0.387 (.503) * |
| dS | Skewness | 1.91 (.055) | 2.43 (.082) | 1.07 (.254) |
|  | Kurtosis | 15.57 (.109) | 17.15 (.164) | 2.44 (.50) |

Group c.

|  |  | Chromosome 1 | Chromosome 2 |
| --- | --- | --- | --- |
| dN | Skewness | 1.20 (.066) | 0.75 (.15) |
|  | Kurtosis | 1.27 (.132) | 0.41 (.30) * |

Group d.

|  |  | *Bordetella* 1 | *Bordetella* 2 | *Burkholderia* 1 | *Burkholderia* 2 |
| --- | --- | --- | --- | --- | --- |
| dN | Skewness | 1.14 (.101) | .799 (.393) * | 1.96 (.101) | 1.45 (.393) |
|  | Kurtosis | 2.45 (.202) | .386 (.768) * | 5.80 (.202) | 1.54 (.768) * |

Group e**.

|  |  | *Vibrio* 1 | *Vibrio* 2 | *Xanthomonas* 1 | *Xanthomonas* 2 |
| --- | --- | --- | --- | --- | --- |
| dN | Skewness | 1.38 (.163) | 1.31 (.616) * | 2.46 (.163) | 3.13 (.616) |
|  | Kurtosis | 2.52 (.324) | 1.37 (1.19) * | 8.98 (.324) | 10.58 (1.19) |

**See comment following Table S8, Group e.
