# Supplementary material for: Why Genes Evolve Faster on Secondary Chromosomes in Bacteria
Source: PLoS Comput Biol. 2010 Apr 1;6(4):e1000732. doi: 10.1371/journal.pcbi.1000732 (PMC2848543; doi:10.1371/journal.pcbi.1000732)
Supplement: Table S7 — Different measures of codon usage bias and predicted expression among genes on different chromosomes (c1-c3). (0.04 MB DOC) [file pcbi.1000732.s009.doc]

Table S7. Different measures of codon usage bias and predicted expression among genes on different chromosomes (c1-c3). A: *Burkholderia cenocepacia* HI2424. B. *Vibrio cholerae* El Tor N16961 All measures were calculated using INCA (30).

A.

| Codon usage measure | C1 | C2 | C3 | F | P |
| --- | --- | --- | --- | --- | --- |
| ENC | 33.7 | 35.4 | 35.7 | 69.6 | <.0001 |
| MILC | 0.810 | 0.862 | 0.861 | 60.2 | <.0001 |
| MELP | 0.720 | 0.679 | 0.661 | 64.0 | <.0001 |
| CAI | 0.650 | 0.609 | 0.600 | 119.0 | <.0001 |
| SCUO | 0.432 | 0.400 | 0.388 | 108.4 | <.0001 |

B.

| Codon usage measure | C1 | C2 | F | P |
| --- | --- | --- | --- | --- |
| MELP | 0.495 | 0.439 | 25.6 | <.0001 |
| CAI | 0.352 | 0.326 | 67.9 | <.0001 |
| SCUO | 0.168 | 0.160 | 9.45 | .002 |
