# Supplementary material for: Why Genes Evolve Faster on Secondary Chromosomes in Bacteria
Source: PLoS Comput Biol. 2010 Apr 1;6(4):e1000732. doi: 10.1371/journal.pcbi.1000732 (PMC2848543; doi:10.1371/journal.pcbi.1000732)
Supplement: Table S8 — Analyses of variance among evolutionary rates of primary and secondary panorthologs shared between Burkholderia and Bordetella. Bordetella dS results were omitted because they are too high to be reliable. (0.04 MB DOC) [file pcbi.1000732.s010.doc]

Table S8. Analyses of variance among evolutionary rates of primary and secondary panorthologs shared between *Burkholderia* and *Bordetella*, as described in Figure 4. Bordetella dS results were omitted because they are too high to be reliable.

|  |  | Sum of squares | df | Mean square | F | Significance |
| --- | --- | --- | --- | --- | --- | --- |
| *Burkholderia* dN x *Burkholderia* chromosome | Between chromosomes | .008 | 1 | .008 | 15.94 | <.0001 |
|  | Within chromosomes | .307 | 617 | .000 |  |  |
|  | total | .315 | 618 |  |  |  |
| *Burkholderia* dS x *Burkholderia* chromosome | Between chromosomes | 1.102 | 1 | 1.102 | 12.687 | <.0001 |
|  | Within chromosomes | 53.58 | 617 | .087 |  |  |
|  | total | 54.68 | 618 |  |  |  |
| *Bordetella* dN x *Burkholderia* chromosome | Between chromosomes | 0.041 | 1 | .041 | 5.466 | 0.02 |
|  | Within chromosomes | 4.577 | 617 | .007 |  |  |
|  | total | 4.617 | 618 |  |  |  |
