# Supplementary material for: Why Genes Evolve Faster on Secondary Chromosomes in Bacteria
Source: PLoS Comput Biol. 2010 Apr 1;6(4):e1000732. doi: 10.1371/journal.pcbi.1000732 (PMC2848543; doi:10.1371/journal.pcbi.1000732)
Supplement: Table S9 — Distribution of panorthologs shared by Burkholderia and Bordetella by chromosome location in Burkholderia and COG annotation. (0.05 MB DOC) [file pcbi.1000732.s011.doc]

Table S9. Distribution of panorthologs shared by *Burkholderia* and *Bordetella* by chromosome location in *Burkholderia* and COG annotation.

| COG | | chromosome | | Total |
| --- | --- | --- | --- | --- |
|  |  | 1.00 | 2.00 |  |
|  | A | 1 | 0 | 1 |
|  | C | 36 | 4 | 40 |
|  | D | 14 | 0 | 14 |
|  | E | 53 | 8 | 61 |
|  | F | 33 | 2 | 35 |
|  | G | 22 | 0 | 22 |
|  | H | 39 | 2 | 41 |
|  | I | 20 | 1 | 21 |
|  | J | 98 | 1 | 99 |
|  | K | 17 | 1 | 18 |
|  | L | 35 | 1 | 36 |
|  | M | 24 | 1 | 25 |
|  | N | 7 | 0 | 7 |
|  | O | 28 | 1 | 29 |
|  | P | 13 | 1 | 14 |
|  | Q | 8 | 0 | 8 |
|  | R | 51 | 8 | 59 |
|  | S | 40 | 5 | 45 |
|  | T | 10 | 0 | 10 |
|  | U | 17 | 0 | 17 |
|  | V | 3 | 0 | 3 |
| Total | | 583 | 36 | 619 |
