# Supplementary material for: Why Genes Evolve Faster on Secondary Chromosomes in Bacteria
Source: PLoS Comput Biol. 2010 Apr 1;6(4):e1000732. doi: 10.1371/journal.pcbi.1000732 (PMC2848543; doi:10.1371/journal.pcbi.1000732)
Supplement: Table S10 — Analyses of variance of the rates of nonsynonymous substitutions among primary and secondary panorthologs shared between Vibrio and Xanthamonas. Estimates of rates of synonymous substitutions were omitted because they are too high to be considered reliable. (0.04 MB DOC) [file pcbi.1000732.s012.doc]

Table S10. Analyses of variance of the rates of nonsynonymous substitution (dN) of primary and secondary panorthologs shared between *Vibrio* and *Xanthamonas*. This analysis was conducted by including the *V. fischeri* ES114 genome as an intermediate genome between the *Vibrio* and *Xanthamonas* groups to facilitate more tolerant alignments and to include more panortholog families for analysis. 236 gene families and only 15 families on the second *Vibrio* chromosome were analyzed here; lacking *V. fischeri*, only 99 families (4 on second chromosome) would have been included for analysis. Estimates of rates of synonymous substitutions were omitted because they are too high to be considered reliable.

|  |  | Sum of squares | df | Mean square | F | Significance |
| --- | --- | --- | --- | --- | --- | --- |
| *Vibrio* dN x *Vibrio* chromosome | Between chromosomes | .204 | 1 | .204 | 16.63 | <.0001 |
|  | Within chromosomes | 2.887 | 235 | .012 |  |  |
|  | total | 3.092 | 236 |  |  |  |
|  | | | | | | |
| *Xanthamonas* dN x *Xanthamonas* chromosome | Between chromosomes | .003 | 1 | .0032 | 2.911 | 0.089 |
|  | Within chromosomes | .262 | 235 | .0011 |  |  |
|  | total | .265 | 236 |  |  |  |
